# Multiple cystic echinococcosis in abdominal and pelvic cavity treated by surgery with a 4-year follow-up: a case report

**DOI:** 10.3389/fmed.2024.1276850

**Published:** 2024-01-18

**Authors:** Lerong Yan, Zhiqiang Chu, Jian Yang, Yongguo Zhang, Guisheng Liu, Zhen Lei, Qian Chen, Jiang Li, Jing Yang, Meifeng Zhao, Shijie Zhang, Xiangwei Wu, Xinyu Peng, Hongwei Zhang

**Affiliations:** ^1^Department of Heaptobiliary Surgery, The First Affiliated Hospital of Shihezi University, Shihezi, China; ^2^School of Medicine of Shihezi University, Shihezi, China; ^3^NHC Key Laboratory of Prevention and Treatment of Central Asia High Incidence Diseases, Shihezi, China; ^4^Xinjiang Production and Construction Corps Infectious Diseases Clinical Medical Research Center, Shihezi, China

**Keywords:** hepatic cystic echinococcosis, pericystectomy, endocystectomy, ascites, bile leakage, negative pressure drainage

## Abstract

We report a case of a male patient who presented with multiple abdominal and pelvic echinococcosis. The patient had been diagnosed with hepatic echinococcosis for 7 years and developed intermittent distension and discomfort in the upper abdomen after an accidental fall. In recent years, the patient’s abdominal distention increased gradually. Computed tomography revealed multiple hydatid cysts in the liver, spleen, abdominal cavity, and pelvic cavity. Abdominal organs were severely compressed, such that he could not eat normally except for a liquid diet. The patient underwent radical surgical resection based on the multi-disciplinary treatment (MDT) and the operation lasted 10 h, nearly 100 hydatid cysts were excised, about 18 liters of cyst fluid and cyst contents were removed, and the patient lost 20 kg of weight after surgery. The operation was successful, but there were still some postoperative complications such as hypovolemic shock, postoperative ascites, postoperative bile leakage. Treatment measures for the patient were anti-infection, antishock, clamping the abdominal drainage tube, and negative pressure abdominal puncture drainage. At follow up the patient’s quality of life had been significantly improved with 15 kg weight gain compared to before.

## 1 Introduction

Cystic echinococcosis (CE) is a complex, chronic, zoonotic infection caused by *Echinococcus granulosus* with a worldwide distribution. It most frequently affects the liver, representing approximately 70% of cases (1–4). Surgery has been the mainstay therapy for hydatid cysts (5, 6). However, hepatic hydatid cyst degeneration, trauma, or response to therapy could lead to the rupture of hydatid cysts. This causes *Echinococcus granulosus* to spread to the biliary tract, abdominal and pelvic cavities, and other adjacent organs, causing complex situations (7). For these complicated cases with multiple cysts in both pelvic and abdominal cavities or involving multiple organs, there are no recommended treatment protocols and few related case reports because of its rarity, the difficulty of surgical treatment, and the uncertainty of therapeutic effects. Many patients gradually become weak and die of exhaustion due to organ compression, difficulty eating, or malnutrition. Resecting multiple hydatid cysts surgically and maximizing patient benefits remain difficult. Surgeons should carefully consider what decisions to make. We report a case of multiple cysts in the abdominal and pelvic cavities, successfully cured by surgical resection in November 2019, to discuss and provide a reference for handling such complex cases.

## 2 Case report

### 2.1 General description

A 35-year-old man had a 7-year history of discontinuous epigastric pain. He visited a local hospital and was diagnosed with hepatic echinococcosis by a B-ultrasound examination. The patient had taken Albendazole before he came to our hospital, but the symptoms had not decreased. Recently, the patient was referred to our hospital for further treatment with a distended abdomen, such that he could not eat normally except for a liquid diet.

The patient had no family history of cystic echinococcosis or other diseases, and no psychosocial history.

He was observed to have a significantly distended abdomen without abdominal wall varicosis. Epigastrium tenderness, drum sound produced by the whole abdominal percussion, unclear liver boundary, percussion pain in the hepatic region, and slowing bowel was observed upon physical examination.

The patient’s blood sample was collected for coagulation function tests, complete blood count, liver and kidney function, and tumor marker detection; no obvious abnormalities were observed (Table 1).

**Table 1:** Preoperative and postoperative laboratory examination of the patient.

Laboratory examination	Results		Unit	Normal range
	Preoperative	Postoperative		
PT	13.70	15.20	Seconds	11.10 ~ 14.20
APTT	38.70	27.60	Seconds	24.00 ~ 32.80
TT	19.90	17.90	Seconds	11.30 ~ 21.90
Fibrinogen	4.66	3.01	g/L	2.00 ~ 4.00
FDP	/	24.38	μg/mL	<5.00
D-Dimer	/	6.12	mg/L	0 ~ 0.55
Total WBC count	5.80	14.50	×10^9^ cells/L	3.50 ~ 9.50
Total RBC count	5.35	5.91	×10^12^ cells/L	4.30 ~ 5.80
Platelet count	311	363.00	×10^9^ cells/L	125.00 ~ 350.00
MCV	80.60	79.70	fl	82.00 ~ 100.00
MCH	25.60	25.20	pg	27.00 ~ 34.00
MCHC	318.00	316.00	g/L	316.00 ~ 354.00
Total protein	76.40	48.70	g/L	65.00 ~ 85.00
Albumin	35.00	22.60	g/L	40.00 ~ 55.00
Globulin	41.40	26.10	g/L	20.00 ~ 30.00
Total bilirubin	9.40	68.80	μmol/L	3.00 ~ 22.00
Direct bilirubin	3.00	30.39	μmol/L	0 ~ 5.00
Indirect bilirubin	6.40	14.10	μmol/L	0 ~ 19.00
ALT	16.70	65.40	U/L	21.00 ~ 72.00
AST	16.60	82.00	U/L	15.00 ~ 40.00
ALP	182.00	100.00	U/L	45.00 ~ 125.00
Triglyceride	0.38	/	mmol/L	0 ~ 1.70
Total cholesterol	3.44	/	mmol/L	0 ~ 5.18
LDL	1.83	/	mmol/L	0 ~ 3.37
Prealbumin	112.00	/	mg/L	250.00 ~ 400.00
HDL	1.04	/	mmol/L	1.16 ~ 1.42
Apolipoprotein-A	0.72	/	g/L	1.00 ~ 1.60
Apolipoprotein-B	0.81	/	g/L	0.60 ~ 1.10

PT, prothrombin time; APTT, activated partial thromboplastin time; TT, thrombin time; FDP, fibrinogen degradation products; WBC, white blood cell; RBC, red blood cell; MCV, mean corpuscular volume; MCH, mean corpuscular haemoglobin; MCHC, mean corpuscular haemoglobin concentration; ALT, alanine aminotransferase; AST, aspartate aminotransferase; ALP, alkaline phosphatase; LDL, low density Lipoprotein; HDL, high density lipoprotein.

**Figure 1:**
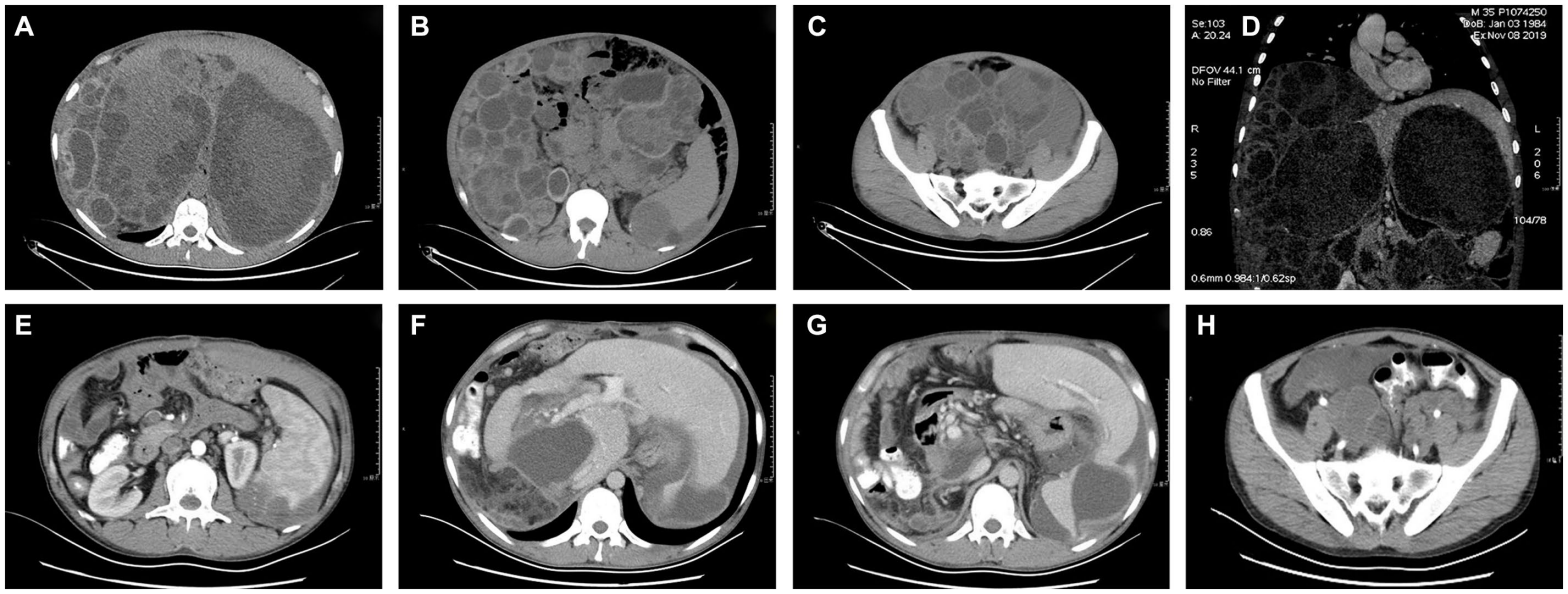
**(A-D)** Preoperative CT images of the pelvis and abdomen. **(E-H)** Postoperative CT images of the pelvis and abdomen.

Computed tomography (CT) revealed multiple hydatid cysts in the liver, spleen, abdominal cavity, and pelvic cavity, and some cyst walls had localized calcification. The portal vein was significantly compressed, and the intrahepatic bile duct of the left lobe of the liver was dilated (Figures 1A–D).

### 2.2 Treatment and progress

Based on the multi-disciplinary treatment (MDT) in consideration of the diagnosis, indications of the operation, the choice of the operation types, and surgical risks, the patient underwent endocystectomy of hepatic hydatid cysts, excision of abdominal hydatid cysts, partial pericystectomy of pelvic hydatid cysts, endocystectomy of pelvic hydatid cysts, lysis of intestinal adhesions on November 18, 2019. In addition, the patient had been taken Albendazole till the operation.

After anesthesia took effect, the patient was placed in a supine position, routine disinfection was performed, and a “J” incision of about 40 cm was made in the upper abdomen. Several hydatid cysts of large sizes with smooth surfaces and oval shapes were found attached to the greater omentum and the liver. Abdominal hydatids were isolated and completely removed one by one to create space for the liver hydatid cyst removal. Several hydatids of different sizes were embedded in the liver and peritoneal wall of the right liver. Right liver atrophy and increased compensation of the left liver were observed. To free the adhesion, high-tension of the right lobe of the liver hydatids were opened and aspirated for decompression. Then the hydatid cyst fluid and endocysts were removed using the Negative Pressure Rotary Cutter (Supplementary Figure S1). The residual cavity was soaked in 20% sterile saline, and the pericystic wall and appendix were partially removed to find the biliary fistula orifices and carefully sutured with 5–0 proline. The same method was applied to the rest of the liver and spleen hydatid cysts. After this, it was applied to the abdominal and pelvic cysts. Complete resection was performed for those in whom it was feasible, and partial resection was performed others after decompression and immersion with 20% saline solution. Before completing the operation, part of the greater omentum was packed into the remnant cavity of the liver hydatid cysts. Negative pressure drainage tubes were placed on the right liver under the diaphragm, in front of the spleen, and the pelvic cavity. The abdomen was closed, and the patient was moved to the ICU for further observation.

**Figure 2:**
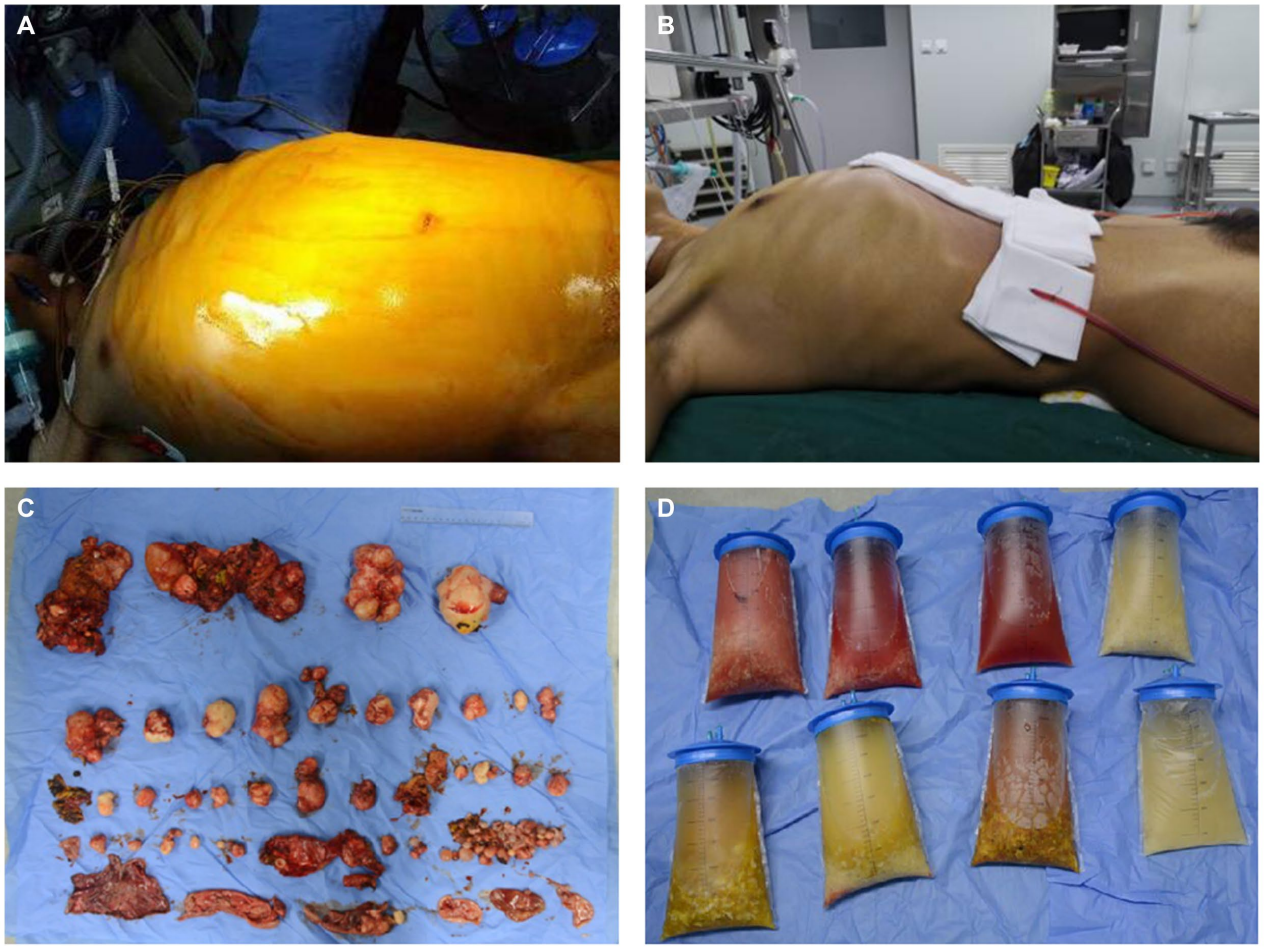
Preoperative and postoperative comparison. **(A)** The preoperative appearance of the patient’s abdomen. **(B)** The postoperative appearance of the patient’s abdomen. **(C)** The hydatid cysts were removed during the operation. **(D)** The hydatid cyst fluid were removed during the operation.

The operation was successful and took more than 10 h, during which approximately 100 hydatid cysts were excised (Figure 2). The patient’s postoperative weight was 20 kg less than before surgery. The postoperative CT scans are shown in Figures 1E–H.

### 2.3 Postoperative problems and corresponding management measures

The patient was given conventional postoperative treatment such as Piperacillin Sodium and Tazobactam Sodium infusion for anti-infection, NSAIDs and glucocorticoids infusion for improving inflammatory responses, Omeprazole infusion for preventing stress ulcers, and Compound Glycyrrhizin and Glutathione infusion for liver functional protection. However, there were still some postoperative problems, and corresponding management measures were as follows.

#### 2.3.1 Hypovolemic shock

The patient developed shock symptoms after the operation, such as sustained hypotension and reduced effective circulating blood volume because of the large number of hydatid cysts in the abdominal and pelvic cavities being removed, which caused a sudden drop of intraperitoneal pressure, and massive loss of body fluid during operation and from the surgical surface. The shock symptoms gradually resolved after a series of anti-shock measures, including adequate fluids reinfusion, noradrenaline and glucocorticoids infusion.

#### 2.3.2 Postoperative ascites

**Figure 3:**
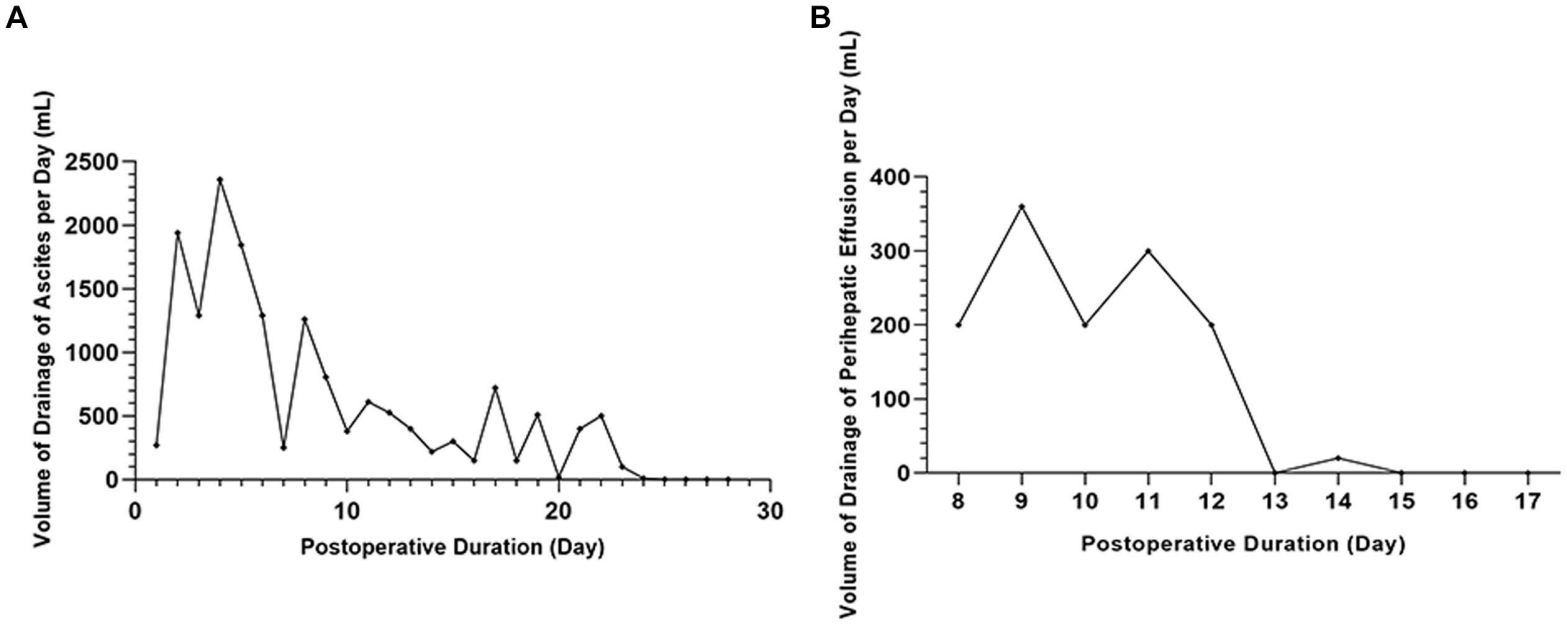
The volume of drainage of ascites and perihepatic fluid. **(A)** No fluid accumulation in the patient’s abdominal cavity by clamping the abdominal drainage tube on the 24th day. **(B)** The bile leakage stopped after one week of treatment with negative pressure suction.

The patient had a large amount of ascites after the operation, which was comprehensively considered transudate according to its appearance and biochemical test. Postoperative drainage continued for 28 days; the total drainage volume during the first 23 days was 17,785 mL, and the volume of drainage of ascites per day was fluctuant but progressively reduced overall during the first 23 days. Albumin and diuretics were used to correct hypoalbuminemia and reduce ascites, but ascites did not disappear. We attempted to clamp the abdominal drainage tube on the 24th day. As a result, it was found that the body could gradually absorb the ascites so that there was no fluid accumulation in the patient’s abdominal cavity (Figure 3A).

#### 2.3.3 Postoperative bile leakage

At postoperative day 8, the right hepatic drainage tube volume gradually reduced. The B ultrasound showed that the drainage tube indwelled on the right liver wound had lost its function. However, perihepatic fluid was detected. Bile was found after a puncture, which indicated bile leakage had occurred. The original drainage tube was removed, and another negative pressure abdominal puncture drainage tube was placed on the liver surgical surface, the deepest part of the fluid. The bile leakage recovered after one week of treatment with negative pressure suction (Figure 3B).

### 2.4 Therapeutic outcome and follow-up result

The CT examination one month after intervention, showed no recurrence or re-occurrence of hydatid cysts in the abdominal and pelvic cavities. The patient recovered well in consideration of stable physical signs. Thus, the patient was discharged after the multidisciplinary discussion on December 31, 2019 and was advised to take albendazole for 3 ~ 6 months and have periodic examinations of liver and kidney function.

There has been no recurrence or re-occurrence of hydatid cysts, given that the patient had a periodic examination at the local hospital in March 2021 (Supplementary Figure S2). At follow up the patient stated that his quality of life had been significantly improved, mental and nutritional status has been well, as well as appetite, and physical capacity increased significantly. Besides, he could currently engage in general physical labor with his 15 kg weight gain compared to before. The latest reexamination was in August last year. Due to the limitations of the condition, he only underwent B-ultrasound examination, which indicated that there were no new hydatid cysts in the abdominal and pelvic cavity, and the remaining hydatid cyst cavity in the liver and spleen was stable, with no signs of recurrence.

## 3 Discussion

### 3.1 Determination of treatment

There are many treatment methods for cystic echinococcosis, and the choice of treatment plan mainly depends on the hydatid cyst classification, existing medical conditions, the experience of surgeons, and whether patients can adhere to long-term treatment and clinical monitoring (8).

Cystic echinococcosis is still managed via surgery currently. There are various surgical methods, among which radical surgery aims to eliminate the hydatid cysts and *Echinococcus granulosus* to achieve the purpose of radical cure (9, 10).

We speculated that the hydatid cysts had ruptured when the patient suffered trauma 7 years earlier, and the hydatid cyst fluid had extravasated into the abdominal cavity, which resulted in that *Echinococcus granulosus* spreading widely and growing in the abdominal and pelvic cavities. The hydatid cysts compressed adjacent organs severely, leading to several complications such as being unable to eat normally, breath restriction. The hydatid cysts grew rapidly because they were very large for the immune system to suppress. Therefore, the patient was soon likely to suffer nutritional disorders, respiratory and circulatory failure, and even death.

The indications of treatment currently provide no recommendations for complicated and multiple cases. Hence, here we listed the treatment options and corresponding possible problems after MDT. (1) Using albendazole alone. The course of the treatment is relatively long, so the patient would soon have complications such as intestinal obstruction and jaundice. In addition, albendazole can often lead to nausea, hepatic injury, and irreversible neutropenia, so the patient must periodically have white blood cell count and liver function tests (5, 11). (2) Percutaneous drainage of hydatid cyst fluid under the guidance of B ultrasound, assisted with albendazole. PAIR (puncture, aspiration, injection, re-aspiration) may temporarily relieve the symptoms of compression caused by a giant hydatid cyst but increase unbearable economic burden (5, 12). However, the residual *Echinococcus granulosus* would continue to proliferate to completely occupy the abdominal and pelvic cavities. PAIR may also lead to several postoperative risks, for instance, bile duct fistula, chemical sclerosing cholangitis, anaphylaxis, or secondary echinococcosis (8). (3) Staging operations. Removing the large hydatid cysts in the liver and abdominal cavity to relieve the symptoms of compression in the 1st stage, radically removing the remaining hydatid cysts in the 2nd stage, with albendazole for adjuvant therapy, which may be intolerant for the patient. (4) To remove the hydatid cysts by radical operation. The risk of this operation might be extremely high, but with a knockdown probability of recurrence (13).

There are complex and diverse interactions between *Echinococcus granulosus* and organisms, which determines that no clinical guidelines could cover all cases. Surgeons should develop personalized treatment based on professional knowledge, experience, and curative effect. Radical surgery should be carried out in most feasible cases, especially for patients with multiple organ echinococcosis. The choice of treatment based on the condition of this case is unprecedented, which provides valuable practical experience for surgical treatment of extremely complex multiple cystic echinococcosis. However, some surgical complications need to be further explored.

### 3.2 Early clamping of the abdominal drainage tube: control of ascites

Drainage of plenty of ascites after the operation caused the poor nutritional status of the patient, which worked against the body restoration. The situation had not improved after fluid infusion, nutritional support, water maintenance, electrolyte and acid–base balance, plasma osmotic pressure maintenance, and human serum albumin transfusion.

The greater omentum is free-hanging mesenteric tissue in the abdominal cavity. It is composed of double peritoneum, which extends downward from the greater curvature of the stomach and the duodenum to the umbilicus or even the pelvis. It covers the front of the small intestine, colon, and other abdominal organs and then folds back to the transverse colon. The overlapping parts usually merge into a four-layered structure rich in blood vessels and fat (14–16). The greater omentum is rich in blood supply; it is supplied by the left and right gastroepiploic arteries, which anastomose in the two layers of the anterior greater omentum along the greater curvature of the stomach and then drain to the portal vein system through the left and right gastroepiploic veins (14). The greater omentum has a large surface area, up to 300 cm^2^ ~ 1,500 cm^2^, and strong extensibility and absorption ability (17).

The greater omentum has a significant absorption effect on normal saline, I^131^-human serum albumin, and I^131^-cholesterol because it is rich in blood vessels, lymphatic vessels, and nerve tissues (18). Therefore, we clamped the abdominal drainage tube on the 24th day with the confirmation of no abdominal infection. There was no fluid accumulation, compression, or infection in the abdominal cavity, and the nutritional status of patients has improved, which can be deduced that the greater omentum could absorb transudate. This might provide experience for solving similar problems in the future.

### 3.3 Tamponade of the greater omentum and negative pressure suction are critically important in the prevention and treatment of residual cavity bile leakage

The greater omentum comprises a trabecular connective tissue framework that carries arteries, veins, lymphatics, and fat pads. Between the trabeculae lie transparent mesothelial membranes. These comprise merely two layers of mesothelial cells that enclose a small connective tissue space and are thin. The stroma may contain scattered fibroblasts, fibrocytes, pericytes, deposits of fat cells, and lymphoreticular bodies (i.e., milky spots) (15). The main functions and clinical applications of the greater omentum are as follows: (1) fat deposition: adipocyte wrapped in the mesothelium, (2) immune function: depending on the milky spot formed by the aggregation of macrophages, (3) limiting the spread of intraperitoneal infection: the greater omentum usually wraps the infected or wound area to limit the spread of infection and promote wound healing (19). In addition, the greater omentum is rich in blood supply, has great extensibility and anti-inflammation ability, and is easy to adhere to other tissues to form many vascular anastomoses, bringing about tissue repair.

Bile leakage is a common postoperative complication of hepatic hydatid cysts (20). One of the key points of the operation is how to dispose of the communication of cysts and biliary tracts. Suturing the bile fistula during operation is efficient prevention, but for some concealed bile fistula or residual cavity with the potential risk of bile leakage, packing with greater omentum can be used for prevention and treatment, which could absorb the liquid in the residual cavity and accelerate the healing of residual cavity and biliary fistula (19, 21, 22). In this case, we used greater omentum packing combined with negative pressure suction to prevent and treat postoperative biliary leakage and obtained significant effect, accumulating relevant experience consequently.

## 4 Conclusion

We made this case report for the purpose of enlightening surgeons and helping other patients with similar problems. It is worth noting that the operation must be carried out in a comprehensive hospital. Having extensive experience in the treatment of hydatid disease is a requirement of surgeons and even entire medical teams. Only if rigorous plans made before the operation, strict accordance with the surgery specifications, and careful postoperative management can maximize the benefits for patients.

## Data availability statement

The raw data supporting the conclusions of this article will be made available by the authors, without undue reservation.

## Ethics statement

Written informed consent was obtained from the individual(s) for the publication of any potentially identifiable images or data included in this article.

## Author contributions

LY: Writing – original draft. ZC: Writing – review & editing. JiaY: Writing – review & editing. YZ: Writing – review & editing. GL: Writing – review & editing. ZL: Writing – review & editing. QC: Writing – review & editing. JL: Writing – review & editing. JinY: Writing – review & editing. MZ: Writing – review & editing. SZ: Writing – review & editing. XW: Writing – review & editing. XP: Writing – review & editing. HZ: Writing – review & editing.
