# Supplementary figures and images for: Multiple cystic echinococcosis in abdominal and pelvic cavity treated by surgery with a 4-year follow-up: a case report
Source: Front Med (Lausanne). 2024 Jan 18;11:1276850. doi: 10.3389/fmed.2024.1276850 (PMC10830638; doi:10.3389/fmed.2024.1276850)

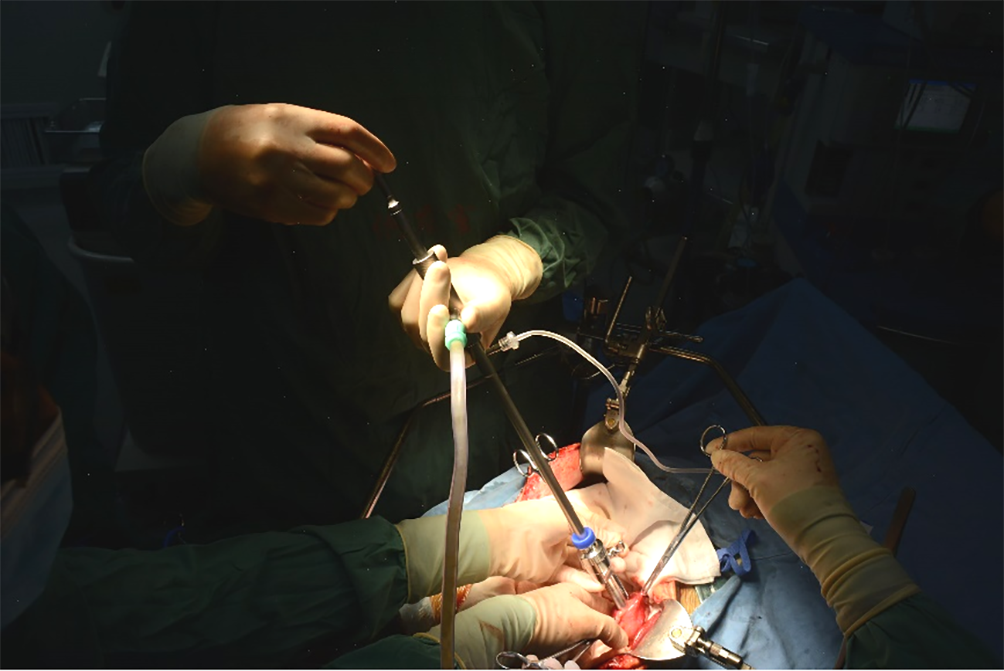

Supplement: Supplementary file 1 [file Image_1.TIF]

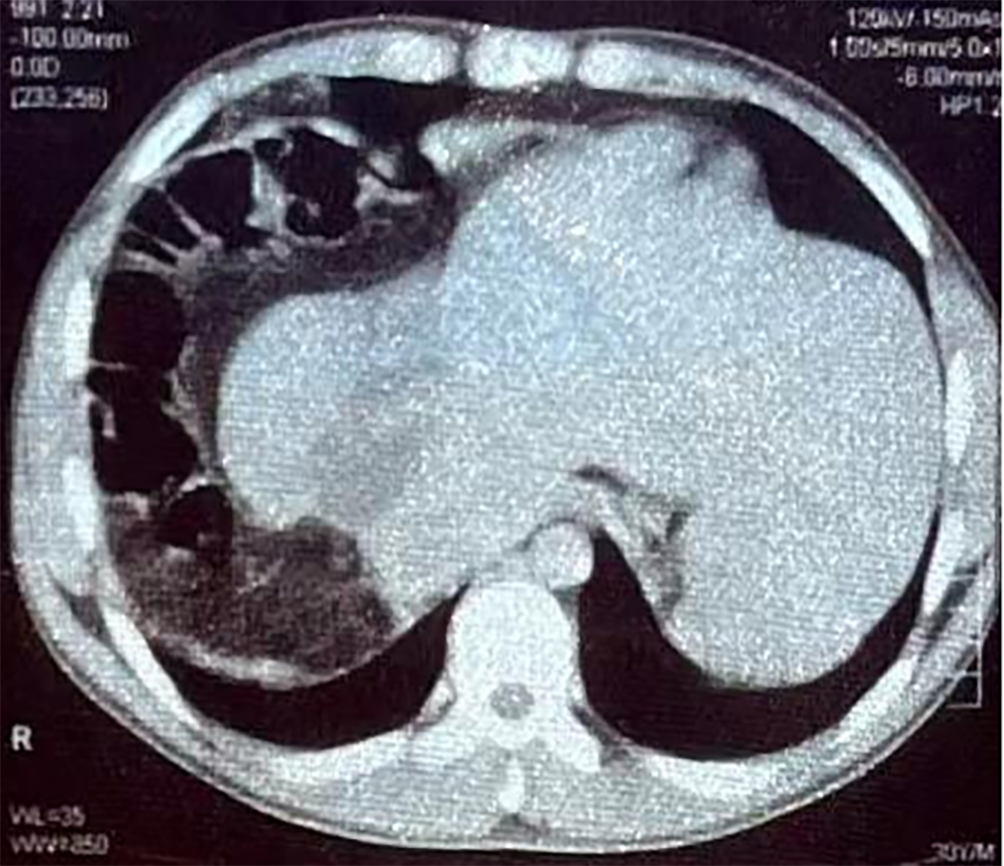

Supplement: Supplementary file 2 [file Image_2.TIF]
